# Non-toxic printed supercapacitors operating in sub-zero conditions

**DOI:** 10.1038/s41598-019-50570-w

**Published:** 2019-10-01

**Authors:** Anna Railanmaa, Suvi Lehtimäki, Jari Keskinen, Donald Lupo

**Affiliations:** Tampere University, Information Technology and Communication Sciences, Korkeakoulunkatu 3, FI-33720 Tampere, Finland

**Keywords:** Electronic devices, Supercapacitors

## Abstract

Aqueous supercapacitors offer a safe alternative for intermediate energy storage in energy harvesting applications, but their performance is limited to relatively warm temperatures. We report the performance of glycerol as a non-toxic anti-freeze for a water-based electrolyte from room temperature to −30 °C at various concentrations. The supercapacitors are manufactured with graphite and activated carbon as current collector and electrode on a flexible polyester (PET) substrate by stencil printing, with a sodium chloride solution as the electrolyte. The devices are characterized at various constant temperatures for electrical performance, as well as in room temperature for mass loss and development of performance over time. It is shown that supercapacitors with glycerol function well in the decreased temperatures compared to water: the capacitance experiences only a slight decrease and the leakage current is significantly reduced. The equivalent series resistance is affected the most by the reduced temperatures, and should be considered the primary limiting factor in low-temperature applications. Electrolytes with 30–40% glycerol perform the best in commercial freezer temperatures, but below −20 °C a higher concentration of 45% glycerol retains better function. The results show great promise for a non-toxic alternative for improving the temperature range of printed supercapacitors.

## Introduction

The Internet of Things (IoT) is one of the most studied topics in recent years, and potential applications range from domestic appliances to industrial equipment and integrated services in our everyday surroundings. One important aspect of IoT is small personal and distributed electronics, which are required to function independently of the power grid or regular battery changes. Energy autonomous sensor and actuator systems can utilize energy from the environment, such as solar or kinetic energy, temporarily store it for later use, and thus enable long-term function without the need for stationary fixtures for power supply or frequent maintenance visits. Supercapacitors^1,2^ are one of the most promising options for this temporary energy storage, as they can be manufactured from inexpensive and safe materials in large scale.

As the demand for distributed intelligence and electronic function in clothes, personal electronics and other everyday applications has increased, it has become evident that in a multitude of applications the ambient temperature can be below the typical room temperature. Use cases such as monitoring food safety of refrigerated or frozen items, ensuring the cold chain of temperature sensitive medicines or any outdoor applications in certain climatic areas require devices to perform reliably all the way to sub-zero conditions. These applications can also require special attention towards user and environmental safety, with strict requisites on materials and packaging, in order to eliminate health risks in case of potential contact between the components and sensitive materials such as food or bare skin. Aqueous salt solutions as the electrolyte are a suitable alternative in printed supercapacitors^3–5^ as well as a safe and affordable alternative for safety critical applications. However, water is not a viable option for sub-zero temperatures on its own, or even with some added salt for electrolyte, and requires an anti-freeze to operate sufficiently.

Organic solvents, such as acetonitrile, propylene carbonate, ethylene glycol and various alcohols and acetates, have been studied for their potential to improve the sub-zero tolerance of supercapacitors^6–11^. Most of these solvents have adverse health effects associated with them, such as irritation or harmfulness if ingested. In applications such as monitoring food safety and wearable electronics, there is always a risk of contamination and thus a risk to the end user. One promising alternative for improving the temperature range is glycerol, which has long been widely used in medical applications, as an additive in food stuffs and in cosmetics, and can be derived from renewable sources (although currently fossil materials are also a common source)^12–15^. It is even endogenously present in the human body, making it a very attractive alternative for safety critical applications in cold environments^16^. As glycerol is a by-product of many industrial processes, it is an inexpensive material for printed electronics, which are aimed to be affordable and easy to produce in mass^15^.

For sub-zero conditions, glycerol is a good candidate for an anti-freeze of water not only because it reduces the freezing point of water significantly^17^, but also because it has a tendency to decrease ice crystal formation, which potentially could be of advantage in activated carbon supercapacitors^18^. Prevention of crystallization may protect the highly porous AC particles from cracking due to crystallization and expansion of water within the pores, which could lead to decreased contact between particles and increase the resistance severely. Additionally, glycerol has a tendency to supercool rather than crystallize both as is and in water solutions, potentially further preventing crystallization^15^.

In this study, we present the performance of a range of glycerol and water solutions with uniform concentration of NaCl as an electrolyte in printed activated carbon supercapacitors. The aqueous solution was selected for safety and to promote ion mobility in the solution, whereas glycerol is incorporated in order to decrease the freezing point and provide better performance in low temperature conditions.

Additionally, glycerol is a good candidate for improving the shelf life of printed aqueous components because of its hygroscopic nature. The effect on shelf life was studied in a drying test with devices prepared on 125 um polyethylene terephthalate (PET) film, which accelerates the experiment due to its relatively weak water barrier. The maximal shelf life can potentially be further improved by advanced packaging methods, as reported previously^19^.

## Experimental

The supercapacitors in this study were prepared by stencil printing on a PET film in laboratory scale. Both the current collector and the activated carbon (AC) electrode were printed with 120 µm stainless steel stencils, with 20 by 30 mm and 18 by 10 mm openings, respectively. First, the current collector was printed with Henkel Electrodag PF407C graphite ink and cured at 120 °C for 15 min in a forced convection oven. The electrode was printed on top of a part of the current collector with an in-house AC ink and dried in ambient air for a minimum of 24 h. The preparation of the ink has been described in more detail by e.g. Lehtimäki *et al*.^3^. The ink consists of AC powder (Kuraray), chitosan powder as binder (Sigma-Aldrich) and a mild acetic acid (Sigma-Aldrich) solution in distilled water. The substrates were weighed before and after printing the AC layer, in order to obtain information of the specific capacitance: the dry weight range was from 5.0 mg to 6.0 mg of AC ink per electrode. Based on the weights of the electrodes, the prints were paired so that the devices were as symmetrical as possible. These pairs were then distributed so that there were samples across the entire weight distribution in each concentration category. The electrolytes were prepared with deionized water, glycerol from Sigma-Aldrich and sodium chloride (NaCl, Sigma-Aldrich).

**Figure 1:**
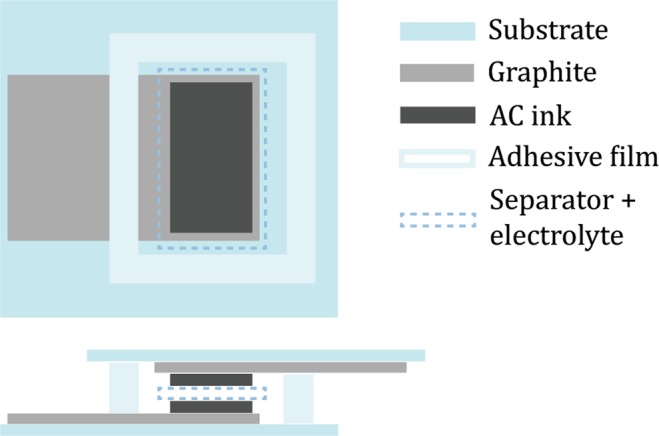
Schematic structure of a single electrode from the top and a cross-section of the entire device. Layer thicknesses are not to scale.

The supercapacitors were assembled in a head-on configuration, as depicted in Fig. 1, showing a cross-section and a top view of the bottom electrode structure. An excess of the electrolyte was pipetted on top of both electrodes, a separator paper (Dreamweaver Silver AR40) was placed on one of the electrodes and it too was soaked with the electrolyte. A frame of double-sided adhesive (3 M 468MP) was placed around the electrode and the second electrode was placed on top, sealing the device while simultaneously pushing any excess air and electrolyte out of the device.

Initial experiments on the electrolyte solutions were conducted in a commercial refrigerator and freezer intended for domestic use. 1 M NaCl solutions with 10 to 90 v-% of glycerol in deionized water were prepared at 10% increments and placed as bulk in vials into the freezer at approx. −19 °C. After allowing the temperatures of the samples to set over night, the solutions with 10–20 v-% of glycerol were determined to be frozen solid and the lower glycerol concentrations were thus left out from further study. Additionally, solutions with concentrations above 60 v-% were found to be extremely viscose at the given temperature and were thus excluded from further study as well. However, 1 M NaCl (aq) was still used as a reference solution in the later measurements to allow for comparison with the formerly used electrolyte solution^3,5,19^. Thus, the concentrations chosen for more detailed testing were 0, 30, 35, 40, 45, 50, 55 and 60% glycerol and supercapacitors with these electrolytes were prepared.

The more detailed study of the chosen concentration range was performed with a Vötsch VT 7010 climate chamber fitted with electrical feed-throughs. The measurements were conducted in ambient moisture and only temperature was controlled by the chamber. The chosen concentrations were first subjected to −30 °C in the Vötsch climate chamber as bulk. After this, the supercapacitor samples were subjected to constant temperature characterization in room temperature of approx. 23 °C, in +10, 0, −10, −20, −25 and −30 °C, and again in room temperature, respectively. At each temperature, the electrical characterization was performed with Maccor 4300 test station (Maccor Inc., USA). There were five samples of each concentration and they were measured in parallel in identical conditions. Additionally, for each glycerol concentration there were three reference samples, which were held in room temperature at all times. The reference samples were measured simultaneously with the low-temperature samples for an equal amount of times, in order to account for the aging in electrical properties due to multiple characterization cycles per device.

The supercapacitors were measured in constant temperature conditions and the temperature was allowed to stabilize for a minimum of 30 minutes after each change, in order to ensure that the samples had fully reached the desired temperature and any potential phase changes had had time to occur. The electrical characterization cycle has been described in great detail before^3,20^; briefly, the cycle consists of constant current charge and discharge sections, with 30 and 60 min holds at 1 V incorporated into the protocol, in accordance with a supercapacitor measurement standard^21^. For full measurement, the cycle was repeated at three different constant current rates (1, 3 and 10 mA) in each temperature. From the measurement protocol, the key characteristics of a supercapacitor were determined: capacitance, equivalent series resistance (ESR) and leakage current. Capacitance was determined from the discharge slope after the 1 h hold of the 1 mA measurement round. ESR was calculated from the IR drop at the beginning of the discharge slope following the 30 min hold period at 10 mA, and leakage current was defined from the current required to maintain the voltage at 1 V at the end of the final one hour hold at the 10 mA measurement. Specific capacitance and leakage were then determined by dividing the absolute values with the total weight of the active layer, since both characteristics are dependent on the mass of the sample, which varies due to the small scale manufacturing technique.

Additionally, the effect of the added glycerol on the possible service life of a printed supercapacitor was estimated through an accelerated aging trial. Supercapacitors with glycerol content of 0, 30, 40, 50 and 60% of the electrolyte were assembled on 125 µm PET sheets. The samples were weighed and electrically characterized once a week with the same measurement protocol as the temperature dependent measurement samples for 15 weeks or until the samples did not function anymore. PET is not an ideal packaging material for long-term storage of aqueous components due to its relatively poor water permeation rate (4 g/m^2^/day for Melinex ST506 at 125 µm). This accelerates the drying of the supercapacitors, which has previously been identified as one of the main causes of failure in printed aqueous supercapacitors over time^4^. The aim was to observe the differences in weight loss and electrical characteristics of the samples with varying electrolyte compositions, rather than aim for maximized service life.

## Results and Discussion

**Figure 2:**
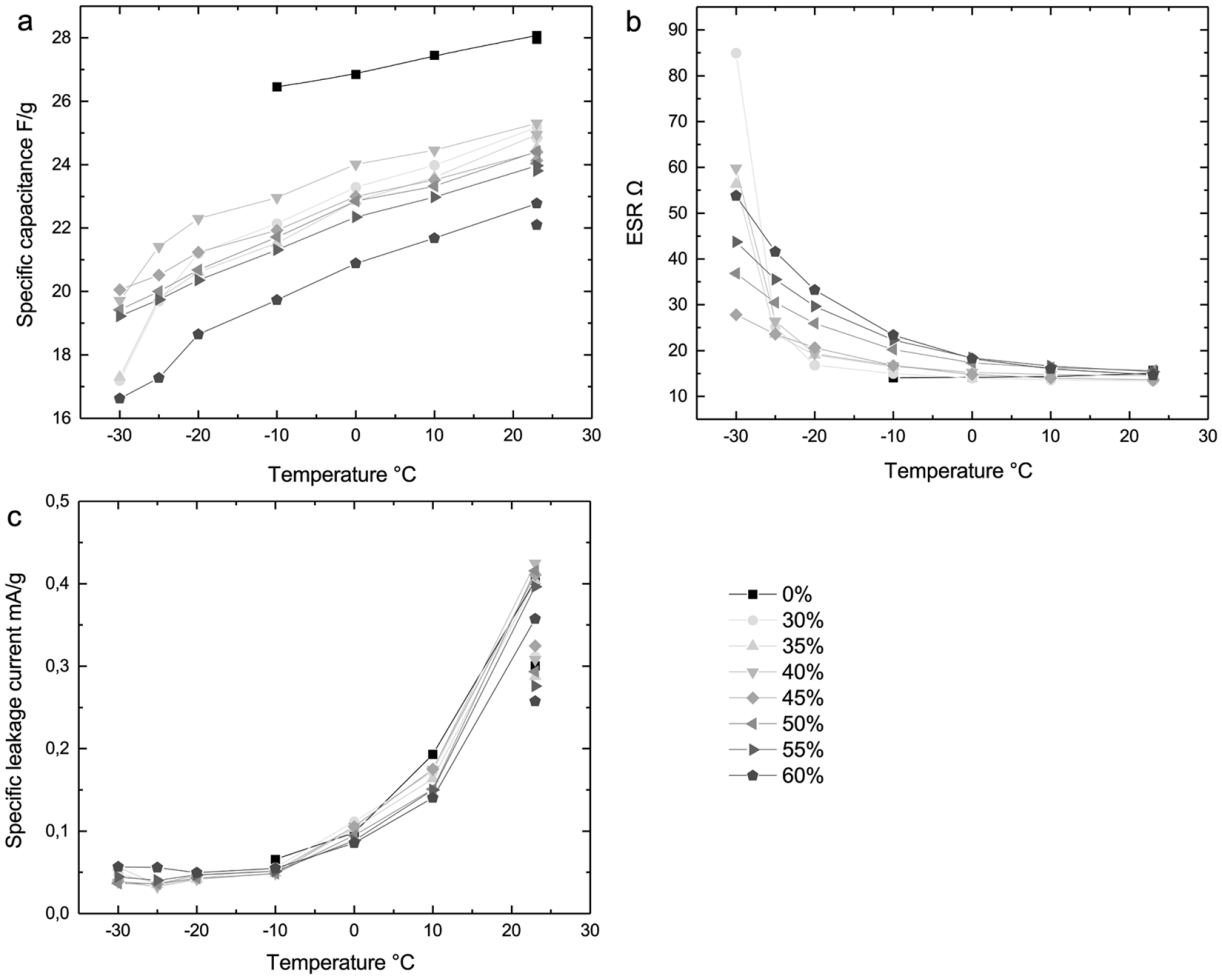
Average performance of temperature test samples in terms of capacitance (**a**), ESR (**b**) and leakage current (**c**). Capacitance and leakage current have been corrected for the weight of AC ink, as they are dependent on the electrode mass. The individual points at room temperature represent the measurement after temperature treatment.

Capacitance, ESR and leakage current are the key performance measures for supercapacitors and these characteristics were measured for the printed devices over the temperature range of +23–−30 °C. Reference samples were measured an equal number of times, in order to separate the effects of aging and decreased temperature form each other, since especially capacitance and leakage current are dependent on the number of measurement cycles^1^. In Fig. 2, the development of the average performance is presented as a function of temperature for each of these characteristics. A more detailed presentation with all individual parallel samples and reference sample performance can be found in supporting Figures S1–S3 in Supporting Information. Some concentrations and temperatures show larger scatter than others, most likely because the samples manufactured by hand are prone to have some variations in assembly and print layers. Exposure to extreme conditions is also likely to emphasize existing differences between samples. However, the variation between samples is quite moderate, considering the harsh conditions and the manufacturing scale.

ESR (Fig. 2b) was found to be the characteristic most variably affected by the reduced temperature. Since ion mobility is dependent on temperature both directly and indirectly through the changing viscosity of the medium, it was expected that the resistance of the electrolyte would be significantly affected by the reduced temperature. Differences between the concentrations arise clearly as the temperature is decreased.

Leakage current (Fig. 2c) is likewise significantly affected by the temperature decrease but most samples react very similarly and all experience a noteworthy decrease in total. On the other hand, capacitance (Fig. 2a) decreases only moderately, likely due to decreased mobility, and different concentrations are affected quite similarly.

It appears that at the coldest temperatures there are two mechanisms at play considering the increasing ESR: one in the dominantly water based solutions and another in the dominantly glycerol ones. At 45% glycerol the increase in ESR at the coldest temperatures is the smallest. Above that concentration, the increasing viscosity of the glycerol solution appears to cause a drastic increase in the resistance and weaken the performance of the devices. On the other hand, the samples with lower glycerol concentrations also experience larger increase in ESR, probably because the high water content exposes the solution to partial freezing and precipitation of water ice, simultaneously increasing the glycerol concentration in the remaining liquid. However, around the typical commercial freezer temperature, approx. −20 °C, the performance of the 30–45% glycerol samples is very similar in terms of ESR, and in these temperatures the lower concentrations can be a viable option e.g. for monitoring food safety and continuity of the cold chain of frozen goods. Regarding the recovery of the supercapacitors from sub-zero temperatures, samples with all concentrations recovered close to their original.

ESR levels when they were returned to room temperature, and no significant effects persist neither from aging, nor from temperature treatment.

It was initially hypothesized that the 1 M purely aqueous solution would be significantly frozen and would not function already at −10 °C based on the water-NaCl phase diagram, where it can be seen that 1 M NaCl (~5, 8 w-%) is located far in the two phase zone of solid water and liquid salt solution^22^. However, it was discovered that the 1 M NaCl supercapacitors were still functional at −10 °C and the experiment was continued to an even lower temperature to confirm failure limits. At −15 °C the devices no longer functioned. Similar observations were made of the 1 M NaCl solutions which had 30–50% glycerol content: the bulk solutions were frozen at −30 °C, but when used in supercapacitors, the devices were still reasonably functional at this temperature. The equivalent series resistance was significantly increased from the value at room temperature, but the samples did still exhibit significant capacitive ability.

**Figure 3:**
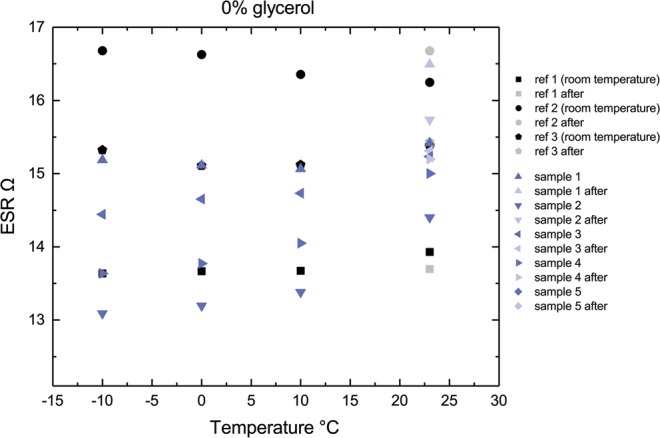
ESR for individual parallel 0% glycerol samples over the temperature range (averaged values in Fig. 2b). It should be noted that the black points represent the reference samples, which have been held at room temperature at all times. However, they are marked to the temperature of the corresponding measurement round to provide reference for the aging of the samples over multiple measurement cycles.

The development of ESR during decreasing temperature is particularly interesting in the case of the 0% glycerol reference solution (Fig. 3), where the ESR appears to decrease, although theoretically both the carbon and graphite conductors as well as the electrolyte should experience an increase in resistivity with decreasing temperature^23,24^.

The contribution of the AC layer and the electrolyte to the ESR has previously been determined to be very small in comparison to the ESR arising from the current collectors^3,4^. Thus, this 5–10% decrease in the resistance could only originate from the current collector, although the conductivity of the graphite particles themselves should also decrease with temperature^23^. This hypothesis was confirmed by measuring the sheet resistances of the current collectors in room temperature and at approx. −19 °C, respectively. It was observed that the 40 °C decrease in temperature produced an average decrease of 0.25 Ohm/□ in the current collectors (the absolute sheet resistance of the samples was approx. 6–8 Ohm/□.) The most likely reason for this is the difference in thermal expansion between the graphite particles and the polymer binder. Typical polymers contract at a significantly higher rate than the graphite^25,26^, pulling the conducting particles closer together and improving the electrical contact in areas where the particles are already connected. Thus, the improving ESR would originate from the reducing contact resistance between the graphite particles and potentially from tunneling across the thinning polymer matrix between the graphite particles^27^. This improvement is then able to slightly counteract the decreasing conductivity of the materials used in this study. In the samples containing a percentage of glycerol, the ESR behaves differently and in the decreased temperatures the ionic conductivity of the electrolyte becomes the limiting factor. In the samples containing glycerol, the clear rise in ESR is attributed to the increasing viscosity of the electrolyte. This change is significant enough to mask any minor positive changes the current collector would be able to produce in these samples.

In general, the changes in capacitance and leakage current are very similar between the different glycerol concentrations, so in practice ESR is clearly the main limiting factor. The capacitance was found to decrease as the temperature decreases and this effect is slightly more pronounced at the highest glycerol concentrations. The specific capacitance values start around 27–25 F/g and decrease to approx. 21–17 F/g at the coldest temperatures, increasing close to the original range when returned to room temperature. The capacitance recovers back to a level which would be expected based on the reference samples, and no permanent effects of temperature treatment appear to remain. The capacitance levels of all samples, including references, are slightly reduced in the “after” measurement in room temperature, most likely due to a reduction in pseudo capacitance^1^ as impurities react non-reversibly causing leakage current, but no major differences appear between references and the temperature samples. A more detailed depiction of capacitance can be found in Figure S1 in the Supplementary Information.

Other organic solvents in aqueous solutions have been reported for their performance as electrolytes in reduced temperatures. Ethylene glycol has been studied with various salts and different electrode compositions; in symmetrical AC supercapacitors capacitances of approximately 22 F/g have been reached at 0 °C^9^. In colder temperatures, with MnO2 and AC electrodes in the supercapacitors, the capacitances at −30 °C varied greatly depending on the electrolyte salt, reaching up to 30 F/g at most, with capacitance highly affected by changes in temperature^10^. Various other alcohols (e.g. methanol, ethanol) have also been studied with sodium perchlorate and at −40 °C have yielded specific capacitances of approximately 15–20 F/g^11^. Overall, it can be observed that sub-zero performance is highly dependent also on the salt and even the electrode materials, so direct comparisons are hard to make.

However, these alternative materials are typically associated with safety concern, which make them unsuitable for many safety critical applications. For example in monitoring the cold chain of temperature sensitive products, such as food or medicines, contamination risk from orally harmful materials (e.g. ethylene glycol, which can cause kidney damage^28^) cannot be tolerated. Glycerol not only has competitive performance but also presents a safe alternative as a non-toxic anti-freeze option.

The leakage current improves, i.e. decreases, significantly as the temperature is decreased, since the main source of leakage current in the devices are Faradaic reactions, whose kinetics are strongly temperature dependent^29^. An overview of the reduction of leakage current can be seen in Fig. 2c and more detailed depictions for each concentration are available in the Supplementary Figure S3. At lower temperatures, the reactions slow down, leading to a lower level of leakage current in total. The slower reaction rate also shows in the room temperature measurements after the temperature treatment as a slightly higher leakage current than what is observed in the reference samples after an equal number of measurement cycles. The reference samples experience a more thorough “burn in” and more of the impurities are able to react during the first few measurement cycles than in the low-temperature samples, where some irreversible reactions have not yet happened due to the lower temperature.

**Figure 4:**
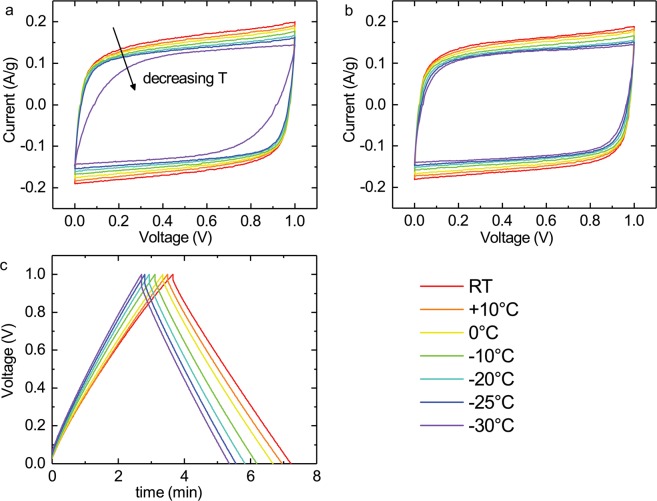
CV curves for solutions with 35% (**a**) and 45% (**b**) glycerol in the electrolyte from room temperature to −30 °C, and an example of the charge-discharge curves over the same temperature range for 45% glycerol (**c**). Plots for other concentrations can be found in the Supplementary Figures S4, S5.

Cyclic voltammogram (CV) plots for select concentrations in all temperatures have been presented in Fig. 4a,b, at scan rate 10 mV/s for a quick general overview. (For all concentrations, please see Figure S4.) The clearest change between temperatures mirrors the formerly observed increase in ESR, as the curve becomes more rounded as temperature is reduced. At lower temperatures the jump in ESR for lower concentrations is clearly observable here as well, whereas higher concentrations progress more steadily. Minor changes in capacitance can be seen in the CV as well as in the constant current measurements in Fig. 2; the reduction is quite moderate across the entire temperature range. It can also be observed that the devices do not exhibit notable levels of pseudocapacitance within this voltage range. Additionally, an example of the charge-discharge curves is presented in Fig. 4c, which shows the same minor shift in capacitance and change in the IR drop indicating the ESR level.

The electrochemical potential window was also briefly studied, since in some research it has been shown that the limited potential window of water can be somewhat affected by introducing additives such as salts and organic solvents to the solution^9,30,31^. Linear sweep voltammetry with Zahner Zennium Electrochemical Workstation from 0 to 3.5 V at 2 mV/s sweep rate was performed on functional devices (see Figure S6 for representative samples at different concentrations over the temperature range). Based on the results, it appears that introduction of glycerol to an aqueous solution does not significantly affect the potential range and the devices are limited to the approx. 1 V of typical aqueous devices.

**Figure 5:**
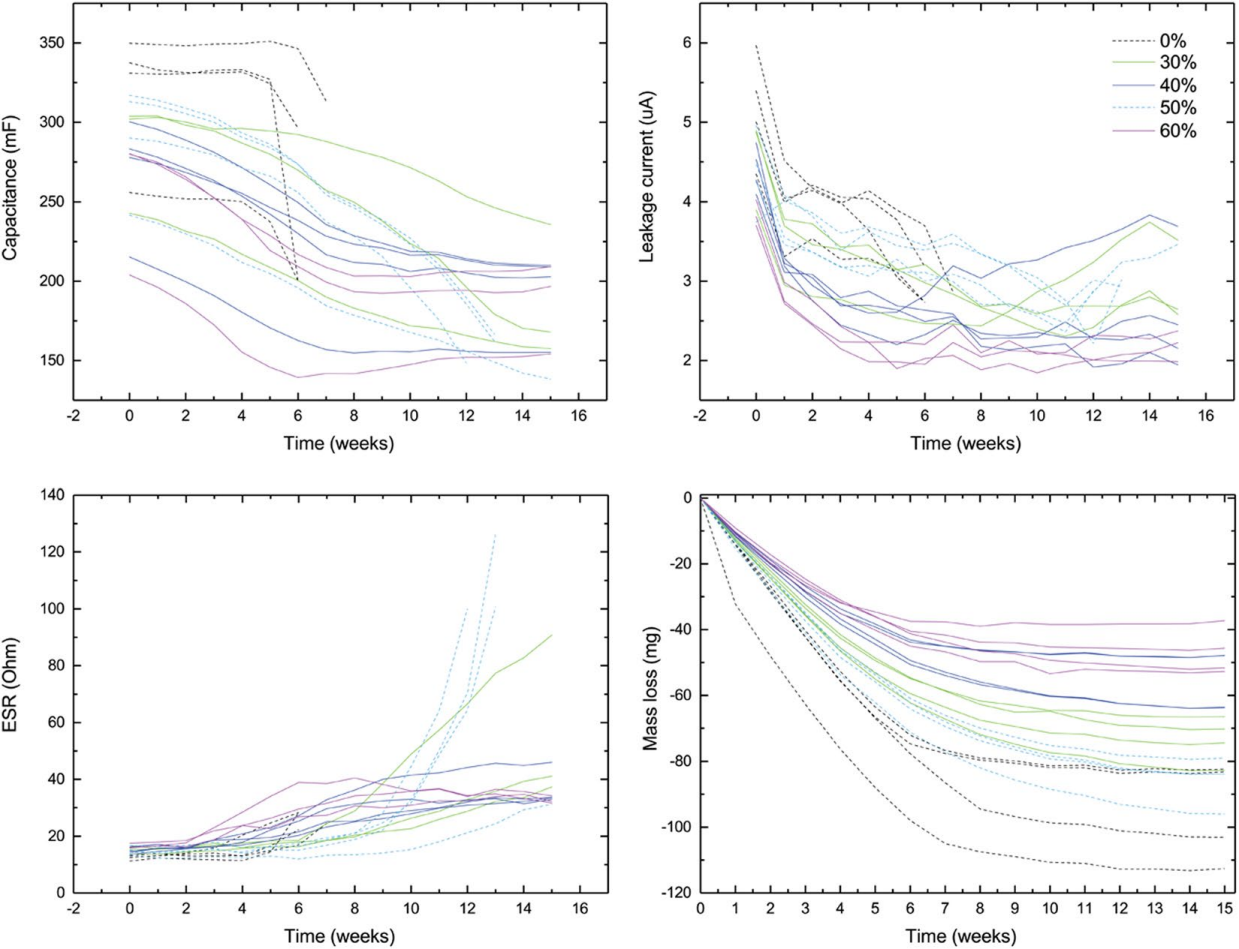
Accelerated drying test, performance of PET packaged supercapacitors. The curves terminate as the device ceases to function.

Additionally, accelerated drying tests were conducted with the glycerol samples in order to estimate the potential lifetime improvements in comparison to a fully aqueous supercapacitor by incorporating a hygroscopic component like glycerol into the device. The test was accelerated by choosing a PET substrate, which is a relatively poor barrier to water, as the packaging material. In terms of the drying behaviour, glycerol significantly affects the potential shelf life of the supercapacitors. Capacitance, ESR, leakage and mass loss from this accelerated study are presented in Fig. 5. The fully aqueous supercapacitors cease to function at week 6–7. There is some weakening in properties just before the complete failure, but still the failure is quite abrupt and before that the effect of aging and drying is somewhat smaller than with the samples containing glycerol. The glycerol samples exhibit a rather steady aging behaviour and their performance develops quite predictably over time. Increase in the ESR is quite steady for most samples as is the decrease in capacitance. This is probably largely related to the changing glycerol concentration in these samples, since most of the mass loss is likely due to water evaporation, leaving behind a higher concentration of glycerol, and leading to the weakening of the performance.

For the most part, the leakage current stabilizes after approximately 8 weeks in samples containing glycerol. Some of the leakage is possibly due to reversible Faradaic reactions of impurities but the final leakage current rate is most likely also dependent on some oxygen permeation through the PET barrier into the device. Just as the general shelf life and drying rate, this could be improved with a less permeable packaging material than in this accelerated test and results do not necessarily represent the best obtainable leakage current level. In total, adding glycerol to the electrolyte solutions appears to be a good option for safely increasing the service life of printed supercapacitors, although some compromising in performance or foot print area might be required.

## Conclusions

In low-temperature trials, the ESR was found to be the main limiting factor in terms of electrical performance. In the coldest temperatures of −25 and −30 °C, the inclusion of 45% glycerol in the solvent was the most advantageous, producing the smallest increase in resistivity. This is of interest especially for outdoor applications in polar regions and at high elevations, where temperature fluctuations can reach these low temperatures naturally. Likely more common application conditions will be at approximately −20 °C which is the typical range of commercial freezers, where the ESR was less of a separating factor and the smaller glycerol concentrations would be preferable for their slightly higher capacitance levels, however the difference being only few percent. Leakage current behaved very similarly across all samples, which is to be expected, as Faradaic reactions are generally known to be heavily temperature dependent.

Glycerol is also effective in decreasing the drying rate of the supercapacitors and is a potential alternative for improving the shelf life of these devices in the future. An accelerated drying test was performed, where glycerol-containing samples functioned for several weeks longer than the samples with only water as the solvent. More research with improved packaging would be required to confirm the maximum effect of the alternative electrolyte, but these results show potential for safely improving the performance.

In total, glycerol offers an excellent option for sub-zero applications, where energy storage is needed but no potential health and safety hazards from toxic chemicals can be allowed. Because of their physical, rather than chemical, energy storage mechanism, supercapacitors are less susceptible to cold temperatures than batteries and they can be manufactured inexpensively and from user safe materials, as has been demonstrated here.

## Supplementary information

Supplementary information
