# Supplementary material for: Non-toxic printed supercapacitors operating in sub-zero conditions
Source: Sci Rep. 2019 Oct 1;9:14059. doi: 10.1038/s41598-019-50570-w (PMC6773738; doi:10.1038/s41598-019-50570-w)
Supplement: Supplementary file 1 — Supplementary information [file 41598_2019_50570_MOESM1_ESM.pdf]

# Supplementary Information

## Non-toxic printed supercapacitors operating in sub-zero conditions

Anna Railanmaa, Suvi Lehtimäki, Jari Keskinen, Donald Lupo

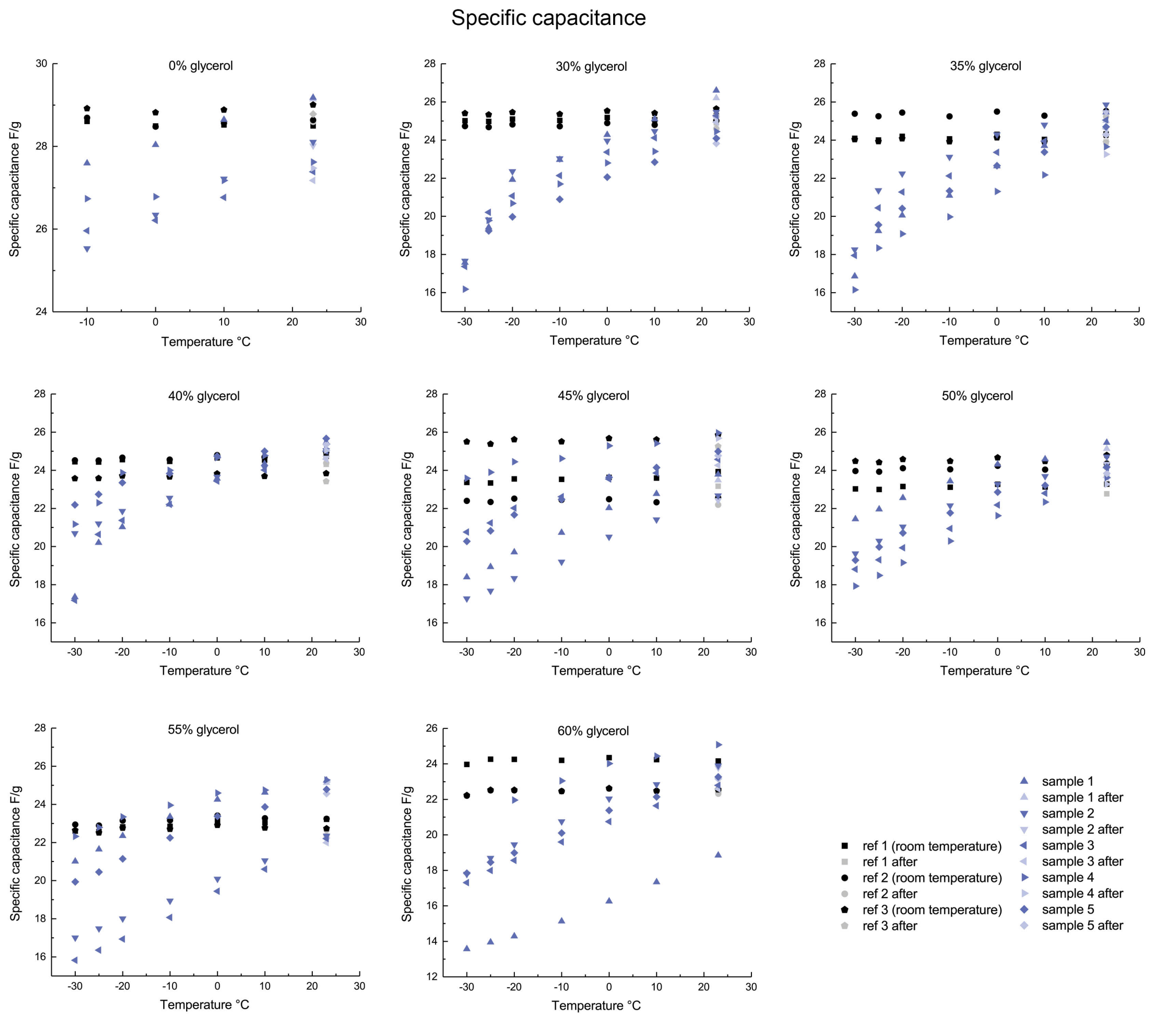


**Figure S1.** Capacitance for all concentrations over the temperature range, including reference samples. References were measured an equal number of cycles but held in room temperature at all times; the data points are marked to the corresponding measurement round.

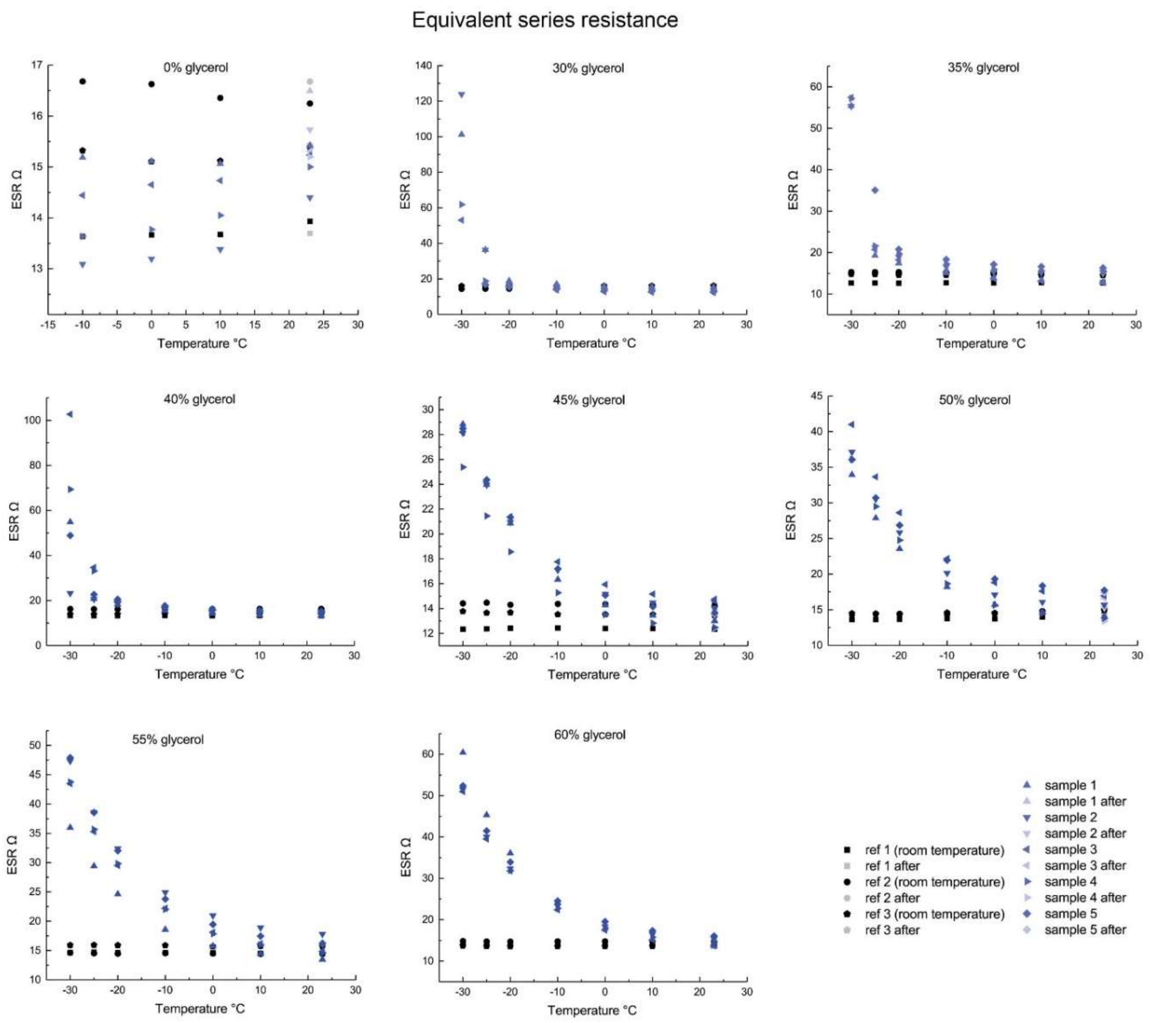


**Figure S2.** ESR for all concentrations over the temperature range, including reference samples. References were measured an equal number of cycles but held in room temperature at all times; the data points are marked to the corresponding measurement round.

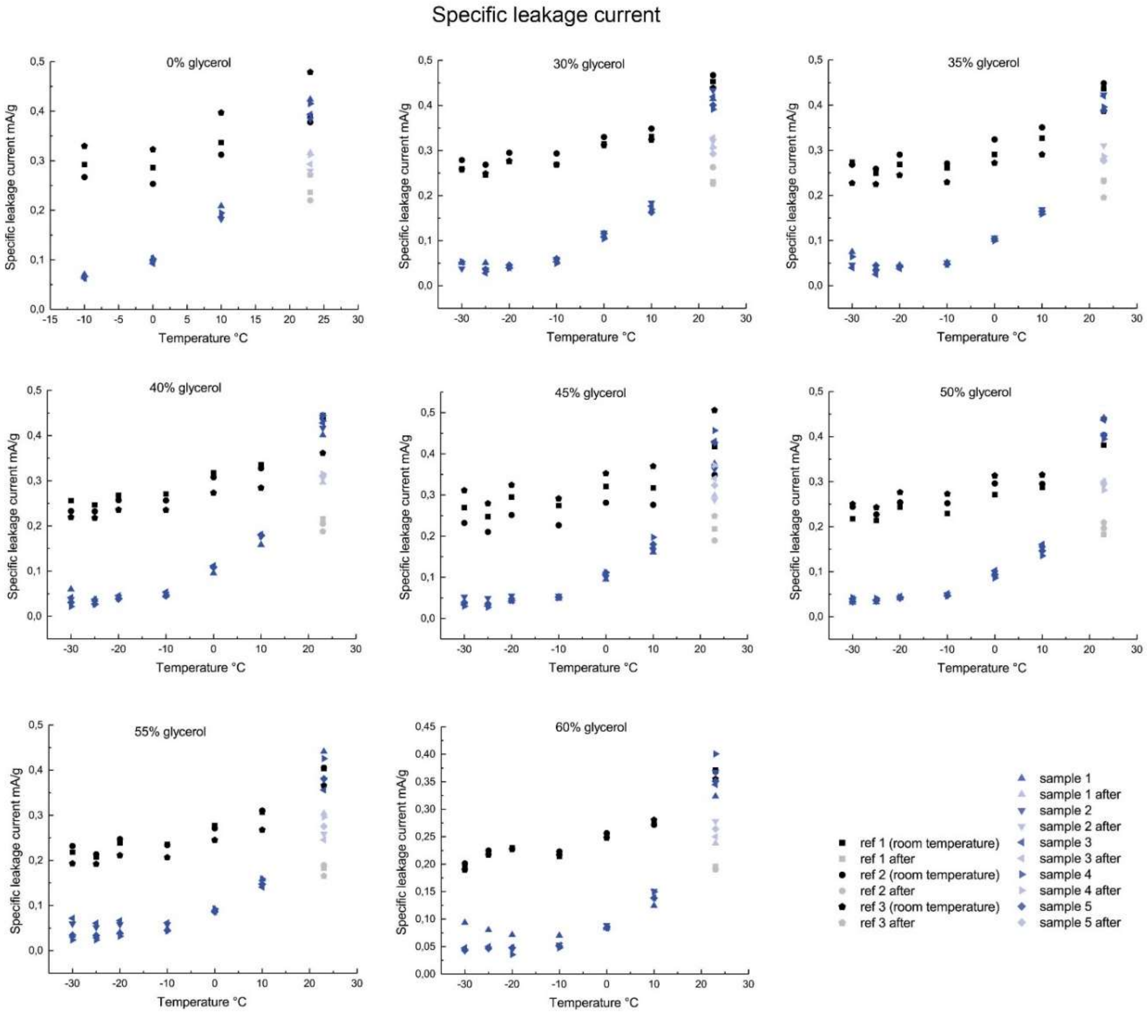


**Figure S3.** Leakage current for all concentrations over the temperature range, including reference samples. References were measured an equal number of cycles but held in room temperature at all times; the data points are marked to the corresponding measurement round.

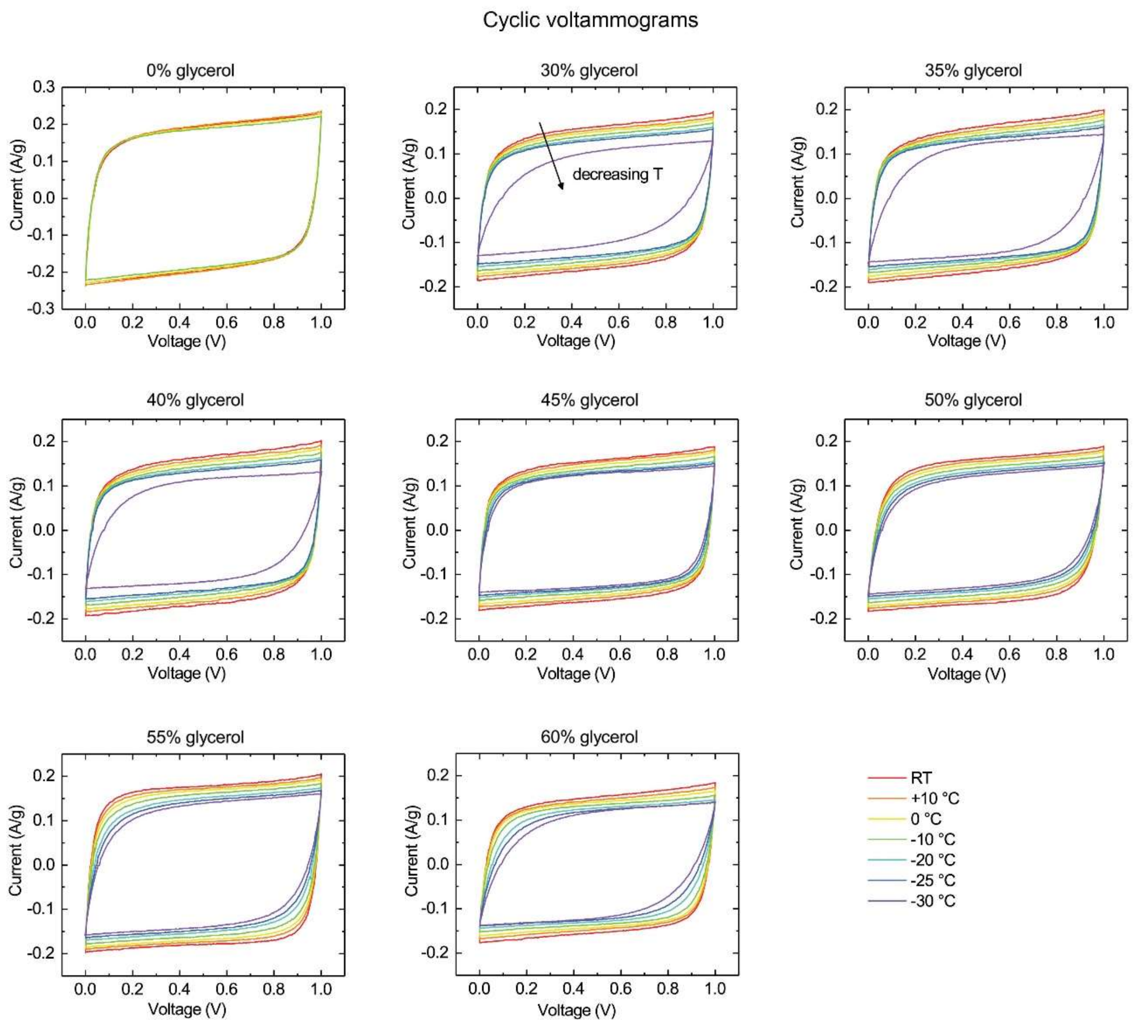


**Figure S4.** Cyclic voltammograms for all concentrations across the temperature range.

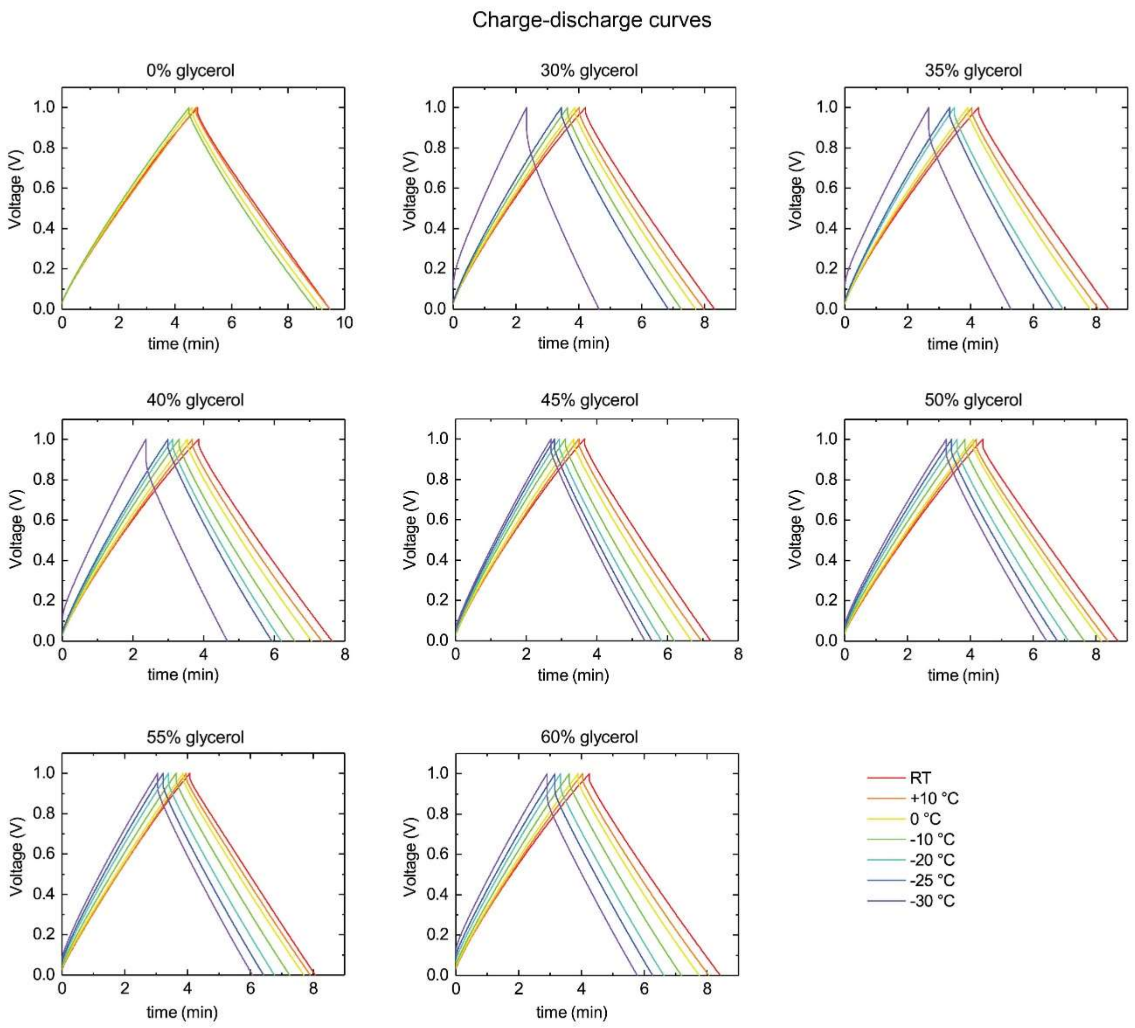


**Figure S5.** Charge-discharge plots for all concentrations across the temperature range.

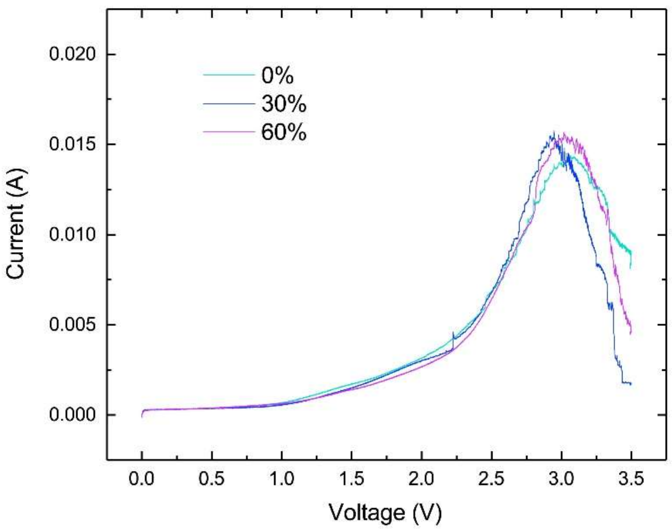


**Figure S6.** Electrochemical stable potential window estimate measured in a symmetrical functional device for the concertation range. All concentrations follow the same trend, which matches water dissociation voltage.
